# Protective Effect of Geraniol on Oxidative, Inflammatory and Apoptotic Alterations in Isoproterenol-Induced Cardiotoxicity: Role of the Keap1/Nrf2/HO-1 and PI3K/Akt/mTOR Pathways

**DOI:** 10.3390/antiox9100977

**Published:** 2020-10-12

**Authors:** Nancy S. Younis, Mohamed S. Abduldaium, Maged E. Mohamed

**Affiliations:** 1 Department of Pharmaceutical Sciences, College of Clinical Pharmacy, King Faisal University, 31982 Al-Ahsa, Saudi Arabia; memohamed@kfu.edu.sa; 2 Pharmacology Department, Zagazig University, Zagazig 44519, Egypt; 3 Department of Cardiology, College of Medicine, Zagazig University, Zagazig 44519, Egypt; Drmohsafwat81@gmail.com; 4 Department of Pharmacognosy, College of Pharmacy, Zagazig University, Zagazig 44519, Egypt

**Keywords:** apoptosis, autophagy, essential oil, monoterpenes, oxidative stress, inflammatory mediators

## Abstract

Background: Myocardial infarction (MI) is still a major contributor to mortality worldwide, and therefore, searching for new drugs is an urgent priority. Natural products are a renewable source for medicinally and pharmacologically active molecules. The objective of this study was to explore the potential of geraniol, a monoterpene alcohol, to protect against MI. Methods: Five groups of Wister rats were used: a control group; a group treated only with geraniol; a group treated only with isoproterenol, to induce MI; and two groups pretreated with geraniol (100 or 200 mg/kg, respectively) for 14 days and challenged with isoproterenol on the 13th and 14th days. Several parameters were measured including electrocardiogram (ECG), cardiac markers, the expression of Kelch-like ECH-associated protein 1 (Keap1), nuclear factor erythroid 2-related factor 2 (Nrf2), and other downstream antioxidant enzymes, as well as the expression of phosphoinositide 3-kinases (PI3K)/protein kinase B (Akt)/mammalian target of rapamycin (mTOR) and other downstream apoptotic and inflammatory mediators. Results: Geraniol treatment reduced the size of the infarct region, attenuated the levels of cardiac indicators, and diminished myocardial necrosis and immune cell infiltration. Geraniol treatment also activated the Keap1/Nrf2/heme oxygenase-1 (HO-1) pathway, increased antioxidant enzyme activities, modulated the PI3K/Akt/mTOR pathway, and ameliorated myocardial autophagy, inflammation, and apoptosis. Conclusion: Geraniol may possess a protective effect against MI through moderating MI-induced myocardial oxidative stress (glutathione (GSH), superoxide dismutase (SOD), glutathione peroxidase (GPx), glutathione S-transferase (GST), and Keap1/Nrf2 pathway), inflammation (IL-1β, IL-6, TNF-α, and Nuclear factor-κB (NF-κB)), apoptosis (caspase-3, caspase-9, Bcl2, and Bax), and autophagy (PI3K/Akt/mTOR pathway).

## 1. Introduction

Cardiovascular diseases (CVDs) are the main cause of mortality globally, contributing to nearly 33% of the total worldwide deaths, with myocardial infarction (MI, heart attack) as a major attributor [1,2].

The tissue damage associated with myocardial infraction has been ascribed to reactive oxygen species (ROS) and numerous related pathways such as tumor protein p53 (p53), AMP-activated protein kinase (AMPK) [3], mitogen-activated protein kinases (p38 kinase, c-Jun N-terminal kinases (JNK) and extracellular signal-regulated protein kinase (ERK1/2)) [1] and Kelch-like ECH-associated protein 1 (Keap1)/nuclear factor erythroid 2-related factor 2 (Nrf2)/heme oxygenase-1 (HO-1). Nrf2 is an oxidation-reduction-sensitive transcription factor that binds to antioxidant response elements (AREs), promoting cell survival gene expression, inhibiting apoptosis, controlling mitochondrial function, and stimulating antioxidant enzyme expression and, therefore, moderating oxidative damage [4]. Under oxidative stress or in response to other damaging stimuli, Nrf2 escapes Keap1-mediated inhibition in the cytoplasm and accumulates in the nucleus, leading to the transcriptional launch of phase II enzymes/antioxidant genes, including HO-1, Glutathione-S-transferase (GST), glutamate-cysteine ligase, catalytic subunit (GCLC), etc. [5]. Li et al. [6] confirmed that Nrf2 pharmacological activation reduced oxidative stress in cardiomyocytes.

Autophagy is a compensatory process that occurs during cellular stress, such as MI; during this process, damaged proteins and cellular organelles are consumed by lysosomes to recycle the nutrients indispensable for keeping cells alive [3]. Within the complex autophagy signaling web, two established pathways have been extensively designated for autophagy regulation [7]; phosphoinositide 3-kinases (PI3K)/protein kinase B (Akt)/mammalian target of rapamycin (mTOR) and AMPK. The Class I PI3K-mTOR signaling pathway is activated in the presence of nutrient enrichment; mTOR activation and mTOR complex (mTORC1) formation via the Akt ultimately prevent formation of the Atg1 complex [7]. The PI3K/Akt/mTOR pathway plays a crucial role in cell growth [8]. Activated Akt regulates cell survival through phosphorylating several downstream targets, such as pro-apoptotic proteins, transcription factors, and endothelial nitric oxide synthase (eNOS) [8]. PI3K and Akt act as connection molecules linking extracellular signals with the cellular reaction, and they play a significant role in regulating the nuclear factor-κB (NF-κB) pathway [9]. The other classical signaling pathway of autophagy is controlled by AMP-activated protein kinase (AMPK), which facilitates autophagy through the regulation of metabolic pathways to protect cardiomyocytes from energy deficiency-induced injury [3,7]. Autophagy has been confirmed to deter apoptosis, suppress the inflammatory and immune reactions, and inhibit the pathogenesis and progression of various diseases in the central nervous system, cardiovascular system (MI, heart failure), endocrine system, digestive system, and others [7].

**Figure 1:**
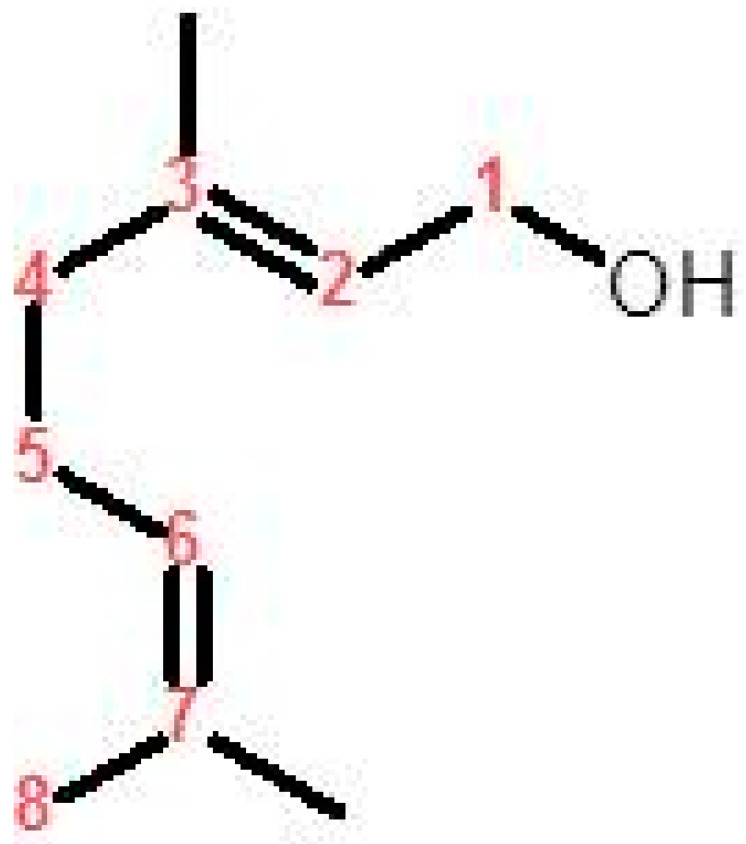
Chemical structure of geraniol.

Medicinal plants, plant extracts, and isolated natural secondary metabolites have conventionally been used to treat numerous clinical diseases, especially those accompanied by oxidative stress [5]. Geraniol (3, 7-dimethylocta-trans-2, 6-dien-1-ol, Figure 1) is an acyclic monoterpene alcohol isolated from the essential oils of numerous aromatic plants including rose (*Rose ottos*) and lemongrass (*Cymbopogon species*) [10,11]. It was approved as GRAS (Generally Recognized as Safe) by the Food and Drug Administration (FDA) to be used as a synthetic flavoring and adjuvant for human consumption [12].

Geraniol was demonstrated to possess numerous pharmacological actions, including neuroprotective [13], antioxidant [13], antinociceptive [14], anti-inflammatory, antifungal [15], anti-arrhythmic [12], anti-osteoarthritis [9], and antitumor [16] activities. In addition, geraniol is a promising drug candidate for diverse health problems such as ulcerative colitis [17], non-alcoholic steatohepatitis [18], hyperlipidemia [19], cardiac complications of diabetes [10], and neuropathic pain [20]. Clinically, dietary geraniol might be an influential constituent as a food supplement for irritable bowel syndrome (IBS) via ameliorating intestinal dysbiosis and relieving IBS symptoms [21]. In addition, in an in vitro model of rotenone-induced Parkinson’s disease, geraniol presented a neuroprotective action by improving antioxidant status and maintaining mitochondrial function [13]. Several lines of experimental evidence addressed the therapeutic or preventive actions of geraniol as well as the underlying mechanisms in diverse types of cancer, such as breast, lung, colon, prostate, pancreatic, and hepatic cancer [16].

This goal of the present study was to appraise geraniol’s actions on isoproterenol-induced MI and to begin dissecting the underlying mechanisms by focusing on two signaling pathways, Keap1/Nrf2/HO-1 and PI3K/Akt/mTOR.

## 2. Materials and Methods

### 2.1. Materials

Geraniol (163333) and isoproterenol (ISO) (16504) were purchased from Merck (Sigma-Aldrich, St. Louis, MO, USA). Colorimetric kits were used to determine antioxidant markers and ATP: glutathione (GSH) (ab65322); superoxide dismutase (SOD) (ab65354); catalase (CAT) (ab83464); glutathione peroxidase (GPx) (ab102530); glutathione S-transferase (GST) (ab65326); and ATP Assay Kit (colorimetric/fluorometric) (ab83355). ELISA kits were used to measure cardiac enzymes (CPK (ab183376), CK-MB (ab187396), cTnT (ab246529) and cTnI (ab246529)); apoptotic markers (cleaved caspase-3 (ab2302) and cleaved caspase-9 (ab2324)); inflammatory mediators (IL-1β (ab100768), IL-10 (ab214566), TNF-α (ab46070) and NF-κB (ab176648)); and Ca^2+^ (o-cresolphthalein complexone (OCPC) (ab146275)). All colorimetric and ELISA kits were obtained from Abcam (Boston, MA, USA), and preformed following the manufacturer’s instructions using a SpectraMax i3x microplate reader (Molecular Devices, San Jose, CA, USA).

### 2.2. Animals and Experimental Design

Wistar rats (male, 200–250 g, 4–6 weeks) were retained with unrestricted access to pellet diet and tap water ad libitum at standard laboratory settings during the whole experiment. All animal procedures were approved by the Animal Research Ethics Committee at King Faisal University under the code number KFU-REC/2019-2-15.

**Figure 2:**
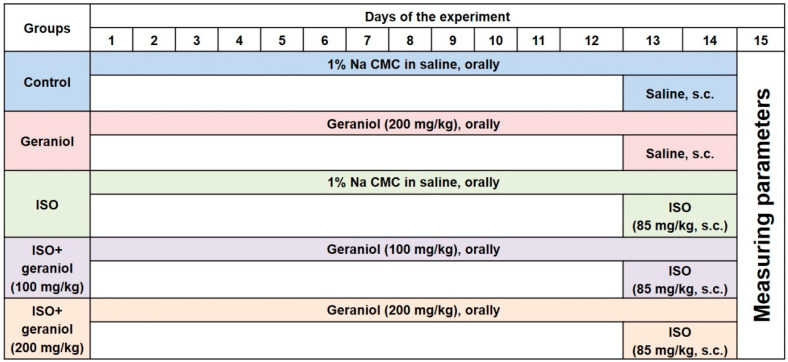
Experimental design showing the different groups and the respective treatments.

The experimental design is illustrated in Figure 2. Animals were randomly divided into five groups (*n* = 6 mice per group). Groups I (control) and II (geraniol) were administered saline orally via gavage or geraniol (orally, 200 mg/kg), respectively, for two weeks and then injected with saline subcutaneously (s.c.) on the last two days of the experiment (days 13 and 14). Group III (ISO) resembled group I, with the difference that the animals were injected (s.c.) with isoproterenol (85 mg/kg) on days 13 and 14 to induce MI. Groups IV (ISO plus geraniol 100) and V (ISO plus geraniol 200) included animals pretreated with geraniol that (either 100 or 200 mg/kg) [10,17] orally via gavage for two weeks and then injected with isoproterenol (85 mg/kg, s.c.) in days 13 and 14 for MI induction [22]. Geraniol (either 100 or 200 mg/kg) was always administered orally via gavage after dissolving in 1% carboxymethyl cellulose (CMC) in normal saline (1 mL).

### 2.3. Electrocardiogram (ECG) Recordings

Animals were anesthetized using urethane (1.5 g/kg) [22] at the end of the experiments and before euthanization, and then placed in a non-invasive computerized ECG apparatus (Kent Scientific, Torrington, CT, USA) to acquire ECG recordings as described previously [22].

### 2.4. Sample Collection

All experimental animals were sacrificed 24 h after the second dose of ISO. Blood samples were collected and centrifuged (15 min, 4000× *g*, 4 °C) to separate the serum, which was frozen at −80 °C for biochemical assays. Hearts obtained from different experimental animals were dissected, weighted, and stored at −80 °C.

### 2.5. Infarct Size Determination

The dissected hearts were cut into four to five transverse sections and incubated in 10% triphenyl tetrazolium chloride (TTC, solubilized in phosphate buffer with pH 7.4, for 30 min in room temperature in the dark). Transverse heart sections were fixed with 10% formalin [5] and then used to calculate the infract area using Image J^®^ (National Institutes of Health, University of Wisconsin, Madison, WI, USA).

### 2.6. Estimation of Antioxidant Markers, ATP, and Ca^2+^ in Heart Mitochondrial Fraction

Mitochondria were isolated from the experimental animals’ heart tissues as previously described [23]. In brief, heart tissues were homogenized in ice-cold 50 mM Tris-Hcl containing 0.25 M sucrose (pH 7.4), and then centrifuged for 20 min at 700× *g*. The supernatant was centrifuged again for 15 min at 9000× *g*, and then the resulting pellets were washed and then suspended in 10 mM of homogenization buffer and stored at −20 °C.

Antioxidant markers (SOD, CAT, GPx, and GST) and the levels of GSH, mitochondrial ATP, and Ca^2+^ were quantified in the previously prepared mitochondria using corresponding assay kits according to the manufactures’ protocols.

### 2.7. Gene Expression Analysis

Real-time PCR was performed as previously described [22]. Quantification analyses were performed in an Opticon-2 Real-time PCR reactor (MJ Research, Reno, NV, USA). Step PE Applied Biosystems (Perkin Elmer, Waltham, MA, USA) software was used to analyze real time-PCR results. The expression of each target gene was measured and adjusted to the expression of β-actin as the reference gene. The primer sequences used in this study are listed in Table 1.

### 2.8. Western Blotting

Western blot analysis to assess protein expression was performed as previously described [23]. Frozen hearts were mixed in RIPA buffer (with protease inhibitor), centrifuged (10,000 rpm, 8 min, 4 °C), and then the supernatant was assessed using a NanoDropLite spectrophotometer (Thermo Fisher Scientific, Austin, TX, USA) to determine the amount of protein. Protein samples (45 µg) were electrophoresed in SDS-PAGE, transported to the polyvinylidene fluoride (PDVF) membrane, incubated with 5% BSA and then with primary antibodies of Keap1 (ab139729), Nrf2 (ab92946), HO-1 (ab189491), PI3K (ab227204), p-PI3K (ab182651), Akt (ab210454), p-Akt (ab38449), mTOR (ab134903), p-mTOR (ab137133) and housekeeping anti-β actin (ab8227), which were obtained from Abcam (Boston, MA, USA) and diluted up to 1:1000. These primary antibodies were identified using horseradish peroxidase (HRP)-conjugated secondary antibodies. Antigen-antibody reactions were visualized with an enhanced chemiluminescence kit (ECL, Sigma-Aldrich) under a gel documentation system. Image J software (National Institutes of Health, University of Wisconsin, Madison, WI, USA) was used to evaluate the acquired images.

### 2.9. Assessment of Inflammatory and Apoptotic Markers

The extracted heart tissues were homogenized in 10% phosphate buffer to be used for the measurement of the apoptotic markers: caspase-3 and caspase-9 and inflammatory mediators: IL-1β, IL-6, TNF-α and NF-κB. ELISA kits were consumed to measure these markers according to the manufacturers’ protocols and using a microplate reader SpectraMax i3x (Molecular Devices).

### 2.10. Histopathological and Immunohistochemical Assays

Heart sections (5 μm-thick) were cut, deparaffinized, dehydrated, and stained with hematoxylin and eosin (H&E) for the evaluation of pathological variations in the heart tissues under light microscopy (Leica DM 300 LED binocular, New York, NY, USA).

Separate heart sections were used for immunohistochemical staining. 3% hydrogen peroxide (H_2_O_2_) in methanol was used to block the endogenous peroxidase enzyme in the obtained sections at 21–25 °C for 30 min, followed by rinsing three times in phosphate-buffered saline (PBS). Afterward, the sections were incubated with NF-κB antibodies (1:100, Thermo Fisher Scientific, Oxford, UK), stored overnight at 4 °C in a humidified chamber, and then goat anti-rabbit-horseradish peroxidase (HRP)-conjugated IgG antibody (1:1000; cat. no. ab6721; Abcam) was added for 1 h at 37 °C. Finally, the sections were developed with 1% diaminobenzidine for 5 min, counterstained with 1% hematoxylin for 2 min at 21–25 °C, and mounted with neutral gum. Heart slices were imagined using a microscope fitted with a digital camera (Nikon Instruments Inc., Tokyo, Japan). NIS-Elements software was used for the quantitative analysis of NFκB. First, the area of the immunohistochemical reaction in the picture was selected. Then, the average optical density in the selected area of each picture was measured. Positive cells were counted under 400× magnification observing 10 consecutive non-overlapping fields per animal in a blinded manner.

### 2.11. Statistical Analysis

Data are presented as mean ± SD. For multiple comparisons, one-way ANOVA followed by Tukey-Kramer as a post-hoc test was performed. *p* < 0.05 was used as the statistical significance level. The ISO group was compared with the control group, and significance was indicated by the symbol “€” in all the relevant figures and tables in the manuscript. The two ISO + geraniol groups were compared to each other and to the ISO group, and significance was indicated by the symbols “*” and “҂”, respectively, in all the relevant figures and tables in the manuscript. All statistical analyses were performed using Prism (GraphPad Sotfware Inc., La Jolla, CA, USA) software, version 5.

## 3. Results

### 3.1. Geraniol Reversed Electrocardiographic Alterations

Electrocardiographic (ECG) constituents including the ST segment, the P wave, the QT, P-R, and R-R intervals, and the QRS complex were electronically recorded. The control group and the geraniol group both displayed normal ECG traces, whereas the ISO-induced MI group showed numerous ECG alterations including a wider ST segment and QT interval, and a shorter P wave, QRS complex, and P-R and R-R intervals compared to controls. Geraniol pretreatment (100 or 200 mg/kg) reversed most of the ECG alterations, as detailed in Table 2.

### 3.2. Geraniol Effect on Infarct Area Size and Heart-to-Body Ratio

**Figure 3:**
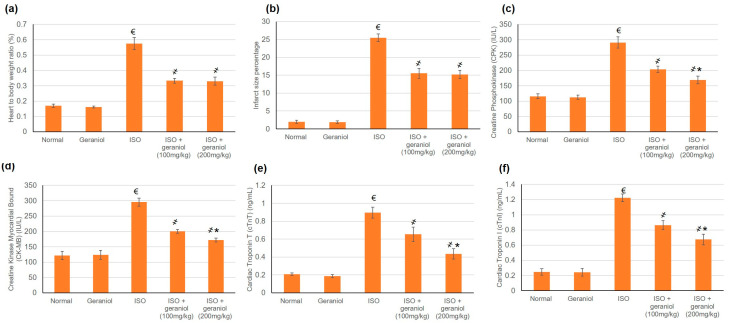
The protective effect of geraniol (100 or 200 mg/kg for 2 weeks) against isoproterenol (ISO)-induced myocardial infraction (MI) as reflected in (**a**) heart-to-body weight ratio, (**b**) infarct area (%), (**c**): creatine phosphokinase (CPK), (**d**) creatine kinase-myocardial bound (CK-MB), (**e**) cardiac troponin T (cTnT), and (**f**) cardiac troponin I (cTnI). All values were stated as mean ± SD (*n* = 6). €, *p* < 0.05 compared to the normal group; ҂, *p* < 0.05 compared to the ISO-induced MI group; *, *p* < 0.05 compared to the ISO + geraniol (100 mg/kg) group. Comparisons done using one-way ANOVA followed by the Tukey-Kramer post-hoc test.

The control group and the geraniol group demonstrated minor infarct areas and normal heart-to-body ratios. In contrast, isoproterenol (ISO)-treated animals disclosed significantly increased heart-to-body ratios together with the presence of infarct areas. Geraniol pretreatment (100 or 200 mg/kg) caused a significant reduction in the infarct areas and heart-to-body ratio compared to the ISO group. There was no significant difference between the two doses of geraniol in infarct area size and heart-to-body ratio as shown in Figure 3a,b, respectively.

### 3.3. Geraniol Effect on Cardiac Enzymes

ISO control animals showed a substantial intensification in numerous cardiac enzymes including Creatine Phosphokinase (CPK), Creatine Kinase Myocardial Bound (CK-MB), Cardiac Tropinine T (cTnT), and Cardiac Tropinine I (cTnI) compared to the control groups, whereas pretreatment with geraniol in both doses—100 and 200 mg/kg—considerably declined these cardiac enzymes, reaching a percentage reduction of 30.2% and 42% for CPK, 32.2% and 41.8% for CK-MB, 21.31% and 51% for cTnT, and 29.4% and 45% for cTnI, as illustrated in Figure 3c–f, respectively.

### 3.4. Geraniol Effect on Mitochondrial Antioxidant Activities

To appraise the role of geraniol on the mitochondrial antioxidant status, the levels of the non-enzymatic antioxidant GSH and the activities of antioxidant enzymes SOD, CAT, GPx, and GST were measured in the various treatment groups (Table 3). The GSH level as well as the activities of mitochondrial antioxidant enzymes were significantly decreased in ISO-challenged animals when compared with controls. As shown in Table 3, geraniol administration (100 or 200 mg/day) increased the GSH levels and the antioxidant enzyme activities.

### 3.5. Effects of Geraniol on the Keap1/Nrf2/HO-1 Pathway

**Figure 4:**
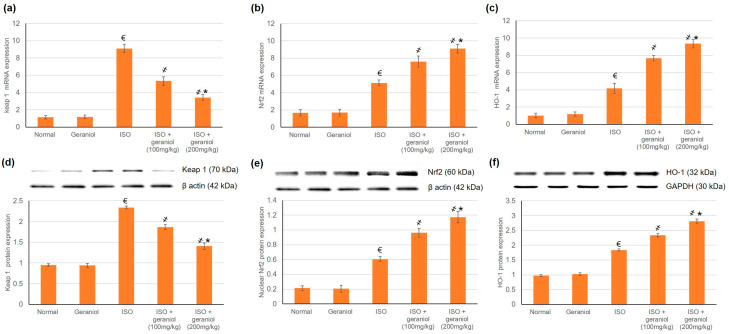
Effects of geraniol pretreatment (100 or 200 mg/kg) on the Keap1/Nrf2/HO-1 pathway in the ISO-induced rat MI model. Gene expression was assessed by determination of the mRNA levels of (**a**) Keap 1, (**b**) Nrf2 and (**c**) HO-1. Protein expression was assessed by determination of the protein levels of (**d**) Keap 1, (**e**) nuclear Nrf2 and (**f**) HO-1 via Western blot analysis. ISO: isoproterenol. All values were stated as mean ± SD (*n* = 6). €, *p* < 0.05 compared to the normal group; ҂, *p* < 0.05 compared to the ISO-induced MI group; *, *p* < 0.05 compared to the ISO + geraniol (100 mg/kg) group. Comparisons done using one-way ANOVA followed by Tukey-Kramer post-hoc test.

To estimate geraniol’s capability to activate Nrf2 signaling in myocardial tissues, both mRNA and protein expression levels of Keap1, Nrf2, and HO-1 were assessed; the results are shown in Figure 4. Challenge with ISO increased the mRNA and protein expression levels of Keap1, Nrf2, and HO-1. Geraniol pretreatment significantly increased the nuclear accumulation of Nrf2 and the mRNA and protein expression levels of HO-1, and it decreased both the mRNA and protein expression levels of Keap1.

### 3.6. Effects of Geraniol on the PI3k/Akt/mTOR Pathway

**Figure 5:**
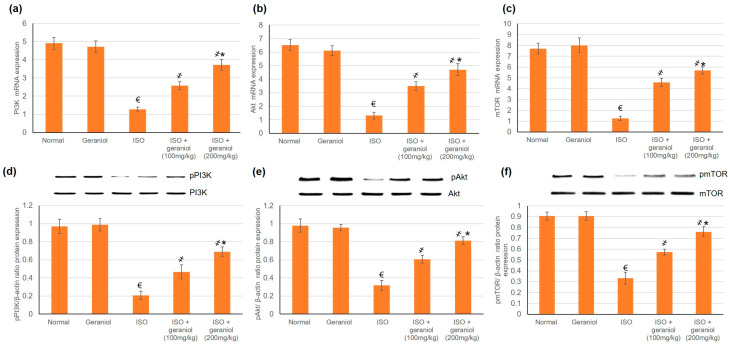
Geraniol (100 or 200 mg/kg) increased the gene and protein expression levels of the PI3k/Akt/mTOR pathway in myocardial infraction rat models. The mRNA expression levels of (**a**) PI3K, (**b**) Akt, and (**c**) mTOR, and the protein expression levels of (**d**) phosphorylated PI3K (pPI3k), (**e**) phosphorylated Akt (pAkt), and (**f**) phosphorylated mTOR (pmTOR) were increased in response to geraniol pretreatment. ISO: isoproterenol. All values were stated as mean ± SD (*n* = 6). €, *p* < 0.05 compared to the normal group; ҂, *p* < 0.05 compared to the ISO-induced MI group; *, *p* < 0.05 compared to the ISO + geraniol (100 mg/kg) group. Comparisons done using one-way ANOVA followed by Tukey-Kramer post-hoc test.

The mRNA and protein expression levels of PI3K, Akt, and mTOR were evaluated, and the results are shown in Figure 5. In the ISO-induced MI group, the protein expression levels of phosphorylated PI3K (p-PI3K), phosphorylated Akt (p-Akt) and phosphorylated mTOR (pmTOR) were significantly decreased. On the other hand, geraniol pretreatment significantly increased the protein expression levels of p-PI3K, p-Akt, and pmTOR in a dose-dependent manner. These findings suggest that geraniol might exert its heart protective function via the activation of PI3k/Akt/mTOR signaling, thereby promoting myocardium autophagy in the ISO-induced MI model.

### 3.7. Geraniol Pretreatement Inhibited Inflammatory Markers

**Figure 6:**
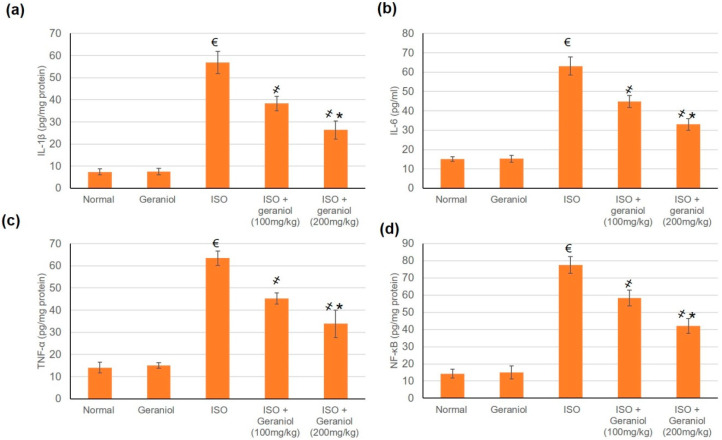
Pretreatment with geraniol (100 or 200 mg/kg) partially suppressed the increase in the levels of inflammatory markers (**a**) IL-1β, (**b**) IL-6, (**c**) TNF-α, and (**d**) NF-κB in response to ISO-induced MI in rats. ISO: isoproterenol. All values were stated as mean ± SD (*n* = 6). €, *p* < 0.05 compared to the normal group; ҂, *p* < 0.05 compared to the ISO-induced MI group; *, *p* < 0.05 compared to the ISO + geraniol (100 mg/kg) group. Comparisons done using one-way ANOVA followed by Tukey-Kramer post-hoc test.

The control group and the geraniol group showed no difference in the levels of inflammatory markers (IL-1β, IL-6, TNF-α, and NF-κB), whereas ISO-induced MI caused a significant elevation of these markers. Pretreatment with geraniol (100 or 200 mg/kg) ameliorated the MI-induced elevation in inflammatory mediators, with respective reductions of 32.57% and 53.64% for IL-1β, 29.04% and 47.73% for IL-6, 28.57% and 46.68% for TNF-α, and 24.67% and 45.74% for NF-κB (Figure 6).

### 3.8. Effects of Geraniol on the Apoptotic Status

**Figure 7:**
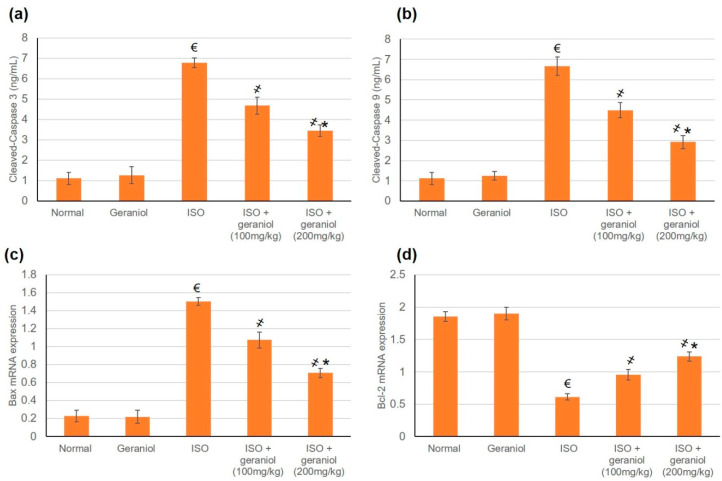
Geraniol pretreatment (100 or 200 mg/kg) partially ameliorated the pro-apoptotic profile observed in isoproterenol (ISO)-induced myocardial infraction (MI). Geraniol ameliorated the protein expression levels of (**a**) cleaved caspase-3 and (**b**) cleaved caspase-9, as well as the mRNA expression levels of (**c**) Bax and (**d**) Bcl2. All values were stated as mean ± SD (*n* = 6). €, *p* < 0.05 compared to the normal group; ҂, *p* < 0.05 compared to the ISO-induced MI group; *, *p* < 0.05 compared to the ISO + geraniol (100 mg/kg) group. Comparisons done using one-way ANOVA followed by Tukey-Kramer post-hoc test.

The apoptotic markers Bax, Bcl2, caspase-3, and caspase-9 showed no differences between the control group and the geraniol group. In the ISO-treated group, the mRNA expression levels of Bax and the protein levels of cleaved caspase-3 and caspase-9 were significantly increased, whereas Bcl-2 mRNA expression levels were decreased, suggesting an apoptotic status within the myocardium of ISO-induced MI animals (Figure 7). However, animals pretreated with geraniol showed ameliorated mRNA expression levels of Bax and protein levels of cleaved caspase-3 and caspase-9, and higher mRNA expression levels of Bcl-2, suggesting that geraniol might limit MI-induced myocardial apoptosis (Figure 7).

### 3.9. Histopathological and Immunohistochemical Assays

**Figure 8:**
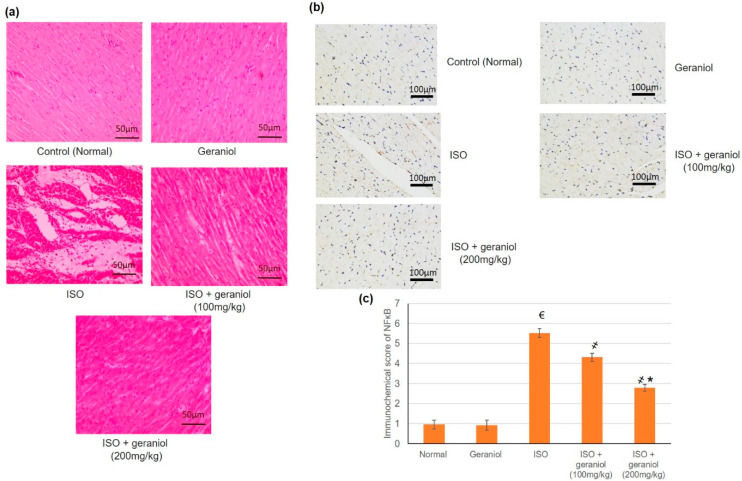
Histopathological investigation (**a**), immunohistochemical assay (**b**,**c**) immunohistochemical scoring of geraniol (100 or 200 mg/kg)-pretreated rats subjected to isoproterenol (ISO)-induced myocardial infraction (MI). Histopathological analysis was performed using hematoxylin and eosin (H&E) staining of the myocardial tissue sections; immunohistochemical staining of cardiac tissues was performed with antibody recognizing NF-κB. All values were stated as mean ± SD (*n* = 6). €, *p* < 0.05 compared to the normal group; ҂, *p* < 0.05 compared to the ISO-induced MI group; *, *p* < 0.05 compared to the ISO + geraniol (100 mg/kg) group. Comparisons done using one-way ANOVA followed by Tukey-Kramer post-hoc test.

In the control and geraniol-treated groups, H&E staining of myocardial tissue sections revealed a regular and clear myocardial structure, with no edema or other signs of inflammation (Figure 8a). On the other hand, animals in the ISO-treated group showed moderate-to-severe myocardial necrosis, edema, and extensive infiltration by immune cells. Animals pretreated with geraniol showed significantly diminished myocardial necrosis as well as reduced edema and immune cells infiltration compared to animals in the ISO-treated group.

Immunohistochemical staining of myocardium samples with an antibody recognizing NF-κB showed no substantial immunoreactivity in the control group and the geraniol group. NF-κB-immunoreactive cells were widely present in the group of ISO-induced MI, whereas animals in the groups pretreated with geraniol showed fewer immunoreactive cells (Figure 8b).

### 3.10. Effects of Geraniol on Heart Mitochondrial Ca^2+^ and ATP Content

**Figure 9:**
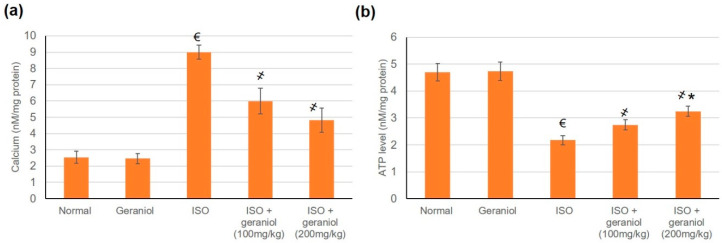
Levels of mitochondrial Ca^2+^ (**a**) and ATP (**b**) in isoproterenol (ISO)-induced myocardial infraction (MI) and effects of geraniol pretreatment. All values were stated as mean ± SD (*n* = 6). €, *p* < 0.05 compared to the normal group; ҂, *p* < 0.05 compared to the ISO-induced MI group; *, *p* < 0.05 compared to the ISO + geraniol (100 mg/kg) group. Comparisons done using one-way ANOVA followed by Tukey-Kramer post-hoc test.

The effects of geraniol on Ca^2+^ and ATP content were evaluated in cardiac cells’ mitochondria. In the group of ISO-induced MI animals, Ca^2+^ levels in cardiac cells’ mitochondrial were significantly increased compared to the control group, whereas mitochondrial ATP levels were decreased. Pretreatment with geraniol ameliorated the Ca^2+^ content and the mitochondrial ATP levels compared with the ISO-treated group (Figure 9).

## 4. Discussion

A deeper understanding of the pathological and biochemical developments in MI has inspired the search for new preventive or therapeutic drugs that could effectively control or cure the myocardial impairment. Essential oils are one of the precious sources for pharmacologically and medicinally active natural compounds. Some essential oils, such as those derived from *Lavandula Angustifolia*, have been used as treatment or protection against many cardiovascular diseases, including ischemic heart disease [24]; however, these oils suffer from qualitative and quantitative alterations in the main components due to environmental and climate factors. The isolation and investigation of the medicinal and pharmacological effects of essential oil components is more useful, and it is current common practice. The present study aimed to characterize the anti-MI properties of geraniol, an essential oil component, and to shed light into its potential mechanisms of action, especially those related to the oxidative stress and apoptosis.

### 4.1. Cardioprotective Effects of Geraniol

The ISO-induced MI group showed numerous ECG alterations, presence of an infarct area, increase in the levels of cardiac enzymes, moderate-to-severe myocardial necrosis, edema, and extensive immune cells infiltration. It has been previously reported that ISO causes detrimental cardiac effects, including necrosis, apoptosis, mitochondrial modifications, oxidative injury, and inflammatory cell infiltration, comparable to what is observed in the ischemic human heart [8,23,25,26]. Compared to ISO-challenged animals, geraniol pretreatment led to a size reduction of the infarct region, attenuation of cardiac indicator enzymes, as well as diminished myocardial necrosis, edema, and immune cells infiltration. All these activities indicated the potential cardioprotective effect of geraniol, prompting investigations of the potential underlying mechanism(s) of action.

Geraniol pretreatment decreased the Ca^2+^ content and increased the ATP content. Earlier reports on the cardiac actions of geraniol by El-Bassossy et al. [27] showed that geraniol could improve the weakened vascular reactivity in diabetes and metabolic syndrome through blocking both voltage-dependent and receptor-operated calcium channels. Furthermore, geraniol proved to exert negative inotropic and chronotropic actions via decreasing both L-type Ca^2+^ and voltage-gated K^+^ currents, acting as a promising anti-arrhythmic candidate [12].

Two main events, which are associated with the MI insult, could contribute to its pathogenesis and the functional deterioration of the cardiac tissue: the increase in ROS levels (oxidative stress) and the induction of apoptosis. Ischemic cells contribute to oxidative stress status through the generation of ROS; meanwhile, the affected cells induce apoptosis in themselves and nearby healthy cells. Therefore, in the current study, pathways related to both events were investigated.

### 4.2. Geraniol and Oxidative Stress: The Keap1/Nrf2 Pathway

The potential antioxidant properties of geraniol to counteract the toxic effects of MI-induced ROS were studied. Specifically, the antioxidant potential of geraniol was investigated through its effect on the Keap1/Nrf2/HO-1 as a redox signaling pathway. Activation of Nrf2 is known to defend against reactive oxidants, while decreased Nrf2 activity has the opposite action [5]. In the current study, challenge with ISO caused the depletion of GSH, reduction of mitochondrial antioxidant enzymes activities, and increase of Keap1 levels, and it decreased Nrf2 and HO-1 mRNA and protein expressions levels, which is indicative of oxidative stress. Former studies demonstrated that ISO administration reduces the activity of mitochondrial antioxidant enzymes leading to cardiac contractile dysfunction and cardiotoxicity, finally resulting in myocardial necrosis [23]. In contrast, geraniol reduced Keap1 expression and increased the nuclear accumulation of Nrf2, which is associated with higher expression levels of HO-1, GSH, and mitochondrial antioxidant enzymes. These results are in accordance with earlier reports suggesting geraniol as an anti-oxidative stress agent. A previous study proved that geraniol (5–200 μM) diminished the endogenous production of ROS in normoxic and hypoxic neonatal rat ventricular cardiomyocytes [1]. Another report proposed that geraniol reduced the levels of the oxidants malondialdehyde and 3-nitrotyrosine and decreased the expression levels of iNOS in animals suffering spinal cord injury [20]. The present study characterized geraniol as a cardioprotective agent in MI, which is possibly associated with the modulation of the Keap1/Nrf2 pathway, enhancing the nuclear accumulation of Nrf2 and the upregulation of phase II antioxidant enzyme expression such as HO-1, SOD, CAT, GPx, and GST.

### 4.3. Geraniol and Autophagy: The PI3K, Akt, mTOR Pathway

Autophagy is a type II cell death mechanism, which is known to be involved in the pathophysiological process of MI; therefore, the modulation of autophagy may be considered as a therapeutic strategy for MI [26]. Previous studies established that compounds that modulate PI3K/Akt signaling can exhibit cardioprotective actions [8,26]. Akt phosphorylation has been found to prevent apoptosis and promote cell survival in the ischemic heart [5]. In the present study, we hypothesized that geraniol might display its cardioprotective efficacy by modifying the PI3K/Akt/mTOR signaling pathway. Hence, the effects of geraniol on mRNA and protein expression levels of PI3K, Akt, and mTOR were investigated as indicators of PI3K/Akt signaling. In ISO-induced MI, the expression levels of pPI3k, pAkt, and pmTOR were decreased, whereas in geraniol-pretreated animals, the levels of these phosphorylated proteins were ameliorated. These results indicate that geraniol caused the activation of PI3k/Akt in a dose-dependent manner and suggest that it may have modulated the myocardial autophagy by this mechanism. In previous studies, numerous drugs have demonstrated cardioprotective activity through activation of the PI3K/Akt pathway; examples include Paeonol and danshensu [5], Vasicine [26], Qi-Li-Qiang-Xin (a traditional Chinese medication) [28], and Levosimendan [29]. Activation of the PI3K/Akt/mTOR pathway also protected against myocardial injury by diminishing oxidative stress, limiting the inflammatory cascade, and deterring apoptosis in vivo and in vitro [8].

### 4.4. Geraniol and Inflammation

MI caused a significant elevation in the expression levels of inflammatory markers, whereas pretreatment with geraniol ameliorated the MI-induced elevation in inflammatory mediators. Geraniol might exert this anti-inflammatory effect either through its modulatory effect on the PI3K/Akt pathway or directly through effects on the inflammatory mediators.

Under normal circumstances, NF-κB binds to IκBα molecules in the cytoplasm in inactive form. When cells are triggered by various chemical or mechanical signals, IκBα is degraded, causing NF-κB heterodimers to enter the nucleus and induce the expression of inflammation-related genes [9]. The PI3K/Akt pathway has been shown to be an upstream element of the NFκB pathway and may also impact the phosphorylation of downstream target proteins [9].

Several previous studies identified a direct inhibitory effect of geraniol on inflammatory mediators. One study showed that geraniol inhibited the nuclear accumulation of NFκB heterodimers [9]. In another study, geraniol improved experimental colitis partly via its antioxidant, anti-inflammatory, and immunosuppressive potentials, possibly by modulating the Wnt/GSK-3β/β-catenin, p38MAPK, NF-κB, and PPARγ signaling pathways [17].

### 4.5. Geraniol and Apoptosis

Bcl-2 is an anti-apoptotic protein that binds to the mitochondrial membrane to prevent cytochrome-C release and inhibit cellular death [26]. On the other hand, Bax is a pro-apoptotic molecule that remains in an inactive conformation in healthy cells. In response to apoptotic stimuli such as MI, Bax undergoes conformational activation and blocks the anti-apoptotic effect of Bcl-2 [26]. In the present study, the mRNA expression levels and protein expression levels of cleaved caspase-3 and caspase-9 were increased in ISO-induced MI, whereas the mRNA expression levels of Bcl-2 were decreased, indicating an apoptotic status within the myocardium. In contrast, geraniol pretreatment ameliorated the observed changes in the levels of Bax, caspase-3, caspase-9, and Bcl-2, indicating cardiac cell protective activity against apoptosis. These findings are consistent with a previous study showing that geraniol administration in the (1-methyl-4-phenyl-1,2,3,6-tetrahydropyridine) MPTP-induced mouse model of Parkinson’s disease controlled the Bcl-2/Bax ratio and prevented the expression of cytochrome C and caspase-9, thereby improving the neurodegeneration state and its functional effects on movement [20].

## 5. Conclusions

The present study highlighted geraniol as a potential cardioprotective agent against ISO-induced MI. Geraniol protected the heart against the MI insult as reflected in the ECG, infarct size, cardiac Ca^2+^, ATP, and marker enzymes (CPK, CK-MB, cTnT, and cTnI). The protective effect of geraniol might be linked to its activity against oxidative stress via the Keap1/Nrf2 pathway modulation and the associated overexpression of the downstream antioxidant enzymes such as HO-1, SOD, CAT, GPx, and GST. Another mechanism contributing to geraniol’s cardioprotective activity might be its regulatory effect of the PI3K/Akt/mTOR signaling cascade, which could control autophagy and apoptosis. Therefore, this study may contribute to the potential future utilization of geraniol as a cardioprotective agent against MI, expanding the medicinal value of this natural monoterpene alcohol.

## Figures and Tables

**Table 1:** Primers sequence used for real-time PCR for gene expression analysis.

TLR Pathway Mediators	Accession Number	Primer Sequence (5′ to 3′)	
		Forward	Reverse
Keap1	NM_057152.2	CTTCGGGGAGGA GGAGTTCT	CGTTCAGATCATCGCGGCTG
Nrf2	NM_031789.2	CATTTGTAGATGACCATGAG TCGC	ATCAGGGGTGGTGAAGACTG
HO-1	NM_012580.2	GTGCACATCGTGCAGAGAA	GTGCACATCCGTGCAGAGAA
PI3K	NM_001371300.1	GCCCAGGCTTACTACAGAC	AAGTAGGGAGGCATCTCG
Akt	NM_033230.2	CGCCTGCCCTTCTACAACC	TCATACACATCTTGCCACACGA
mTOR	NM_019906.1	CTGTAATTACATCCTCGACTG	CGTGTCGTGGTTAGTCG
Bax	NM_017059.2	GTGGTTGCCCTCTTCTACTTTG	CAAAAGATGGTCACTGTCTGC
Bcl-2	NM_016993.1	CCGGGAGATCGTGATGAAGT	ATCCCAGCC TCCGTTATCCT
β-actin	NM_031144.3	CACGATGGAGGGGCCGGACTCATC	TAAAGACCTCTATGCCAACACAGT

**Table 2:** The impact of geraniol pretreatment on ECG components in isoproterenol-induced myocardial infarction.

ECG Component	Control	Geraniol	ISO	ISO + Geraniol (100 mg/kg)	ISO + Geraniol (200 mg/kg)
ST elevation (mV)	0.0267 ± 0.0016	0.0252 ± 0.0018	0.1840 ± 0.0126 €	0.1210 ± 0.0086 ҂	0.0810 ± 0.0066 ҂*
QT interval (sec)	0.0453 ± 0.0037	0.0460 ± 0.0006	0.0933 ± 0.0034 €	0.0743 ± 0.0028 ҂	0.0643 ± 0.0028 ҂*
P Wave (sec)	0.0192 ± 0.0030	0.0190 ± 0.0019	0.0047 ± 0.0005 €	0.0133 ± 0.0008 ҂	0.0163 ± 0.0008 ҂*
QRS complex (sec)	0.0416 ± 0.0008	0.0417 ± 0.0005	0.0231 ± 0.0009 €	0.0305 ± 0.0009 ҂	0.0338 ± 0.0009 ҂*
P-R interval (sec)	0.2450 ± 0.0105	0.2600 ± 0.0167	0.1398 ± 0.0026 €	0.1817 ± 0.0194 ҂	0.2117 ± 0.0194 ҂*
R-R interval (sec)	0.2300 ± 0.0310	0.2237 ± 0.0117	0.1453 ± 0.0008 €	0.1798 ± 0.0023 ҂	0.1983 ± 0.0023 ҂*

All values were stated as mean ± SD (*n* = 6). €, *p* < 0.05 compared to the normal group; ҂, *p* < 0.05 compared to the ISO-induced MI group; *, *p* < 0.05 compared to the ISO + geraniol (100 mg/kg) group. Comparisons done using one-way ANOVA followed by Tukey-Kramer post-hoc test.

**Table 3:** Impact of geraniol pretreatment on GSH levels and mitochondrial antioxidant enzymes activities in isoproterenol-induced myocardial infarction.

Antioxidants	Control	Geraniol	ISO	ISO + Geraniol (100 mg/kg)	ISO + Geraniol (200 mg/kg)
GSH (nmol/mg protein)	7.28 ± 0.24	7.45 ± 0.12	3.66 ± 0.19 €	5.49 ± 0.19 ҂	6.24 ± 0.26 ҂*
SOD (micromol H_2_O_2_/min/mg protein)	124.14 ± 6.25	128.16 ± 5.46	65.43 ± 8.4 €	89.49 ± 4.60 ҂	95.19 ± 6.10 ҂*
CAT (micromol H_2_O_2_/min/mg protein)	1.49 ± 0.06	1.46 ± 0.08	0.64 ± 0.06 €	1.02 ± 0.05 ҂	1.17 ± 0.03 ҂
GPx (µg of GSH utilized/min/mg protein)	19.63 ± 1.4	20.25 ± 2.1	7.46 ± 1.51 €	12.10 ± 1.24 ҂	15.54 ± 2.43 ҂*
GST (nmol/mg protein)	524.2 ± 15.86	532.41 ± 6.71	382.5 ± 12.93 €	457.11 ± 4.24 ҂	483 ± 16.48 ҂*

GSH: glutathione; SOD: superoxide dismutase; CAT: catalase; GPx, glutathione peroxidase; GST: glutathione-S-transferase; All values were stated as mean ± SD (*n* = 6). €, *p* < 0.05 compared to the normal group; ҂, *p* < 0.05 compared to the ISO-induced MI group; *, *p* < 0.05 compared to the ISO + geraniol (100 mg/kg) group. Comparisons done using one-way ANOVA followed by Tukey-Kramer post-hoc test.
